# Combining Site Occupancy, Breeding Population Sizes and Reproductive Success to Calculate Time-Averaged Reproductive Output of Different Habitat Types: An Application to Tricolored Blackbirds

**DOI:** 10.1371/journal.pone.0096980

**Published:** 2014-05-09

**Authors:** Marcel Holyoak, Robert J. Meese, Emily E. Graves

**Affiliations:** 1 Department of Environmental Science and Policy, University of California Davis, Davis, California, United States of America; 2 Department of Wildlife, Fish and Conservation Biology and Avian Sciences Graduate Group, University of California Davis, Davis, California, United States of America; University of Milan, Italy

## Abstract

In metapopulations in which habitat patches vary in quality and occupancy it can be complicated to calculate the net time-averaged contribution to reproduction of particular populations. Surprisingly, few indices have been proposed for this purpose. We combined occupancy, abundance, frequency of occurrence, and reproductive success to determine the net value of different sites through time and applied this method to a bird of conservation concern. The Tricolored Blackbird (*Agelaius tricolor*) has experienced large population declines, is the most colonial songbird in North America, is largely confined to California, and breeds itinerantly in multiple habitat types. It has had chronically low reproductive success in recent years. Although young produced per nest have previously been compared across habitats, no study has simultaneously considered site occupancy and reproductive success. Combining occupancy, abundance, frequency of occurrence, reproductive success and nest failure rate we found that that large colonies in grain fields fail frequently because of nest destruction due to harvest prior to fledging. Consequently, net time-averaged reproductive output is low compared to colonies in non-native Himalayan blackberry or thistles, and native stinging nettles. Cattail marshes have intermediate reproductive output, but their reproductive output might be improved by active management. Harvest of grain-field colonies necessitates either promoting delay of harvest or creating alternative, more secure nesting habitats. Stinging nettle and marsh colonies offer the main potential sources for restoration or native habitat creation. From 2005–2011 breeding site occupancy declined 3x faster than new breeding colonies were formed, indicating a rapid decline in occupancy. Total abundance showed a similar decline. Causes of variation in the value for reproduction of nesting substrates and factors behind continuing population declines merit urgent investigation. The method we employ should be useful in other metapopulation studies for calculating time-averaged reproductive output for different sites.

## Introduction

A common conservation aim is to understand the relative roles of altered habitat characteristics versus fragmentation in population declines. Armstrong [1] stated this as the need to distinguish between the habitat and metapopulation paradigms. Specifically, that we needed to identify how population declines and dynamics are influenced by habitat characteristics (e.g., in species' distribution or niche models [2]), and the metapopulation processes of extinction and colonization [3], [4]). Here we tackle the question of how to evaluate the contribution to long-term regional dynamics of breeding populations in habitat patches of different types when patches do not remain continuously occupied. Our focus is on breeding populations because our study species, the Tricolored Blackbird (*Agelaius tricolor*), is widely dispersed when it is not breeding, and consequently it is difficult to census outside of the breeding season. Spatial concentration of numbers during the breeding season is also observed in a variety of organisms, including various land birds, pond-breeding amphibians and aquatic insects. Additionally in our study species, Tricolored Blackbirds, low breeding success has been highlighted as a problem during 2006–2011 [5]. We calculate a time-averaged index of reproduction that we believe will be of interest to those studying metapopulations of other organisms that do not use the same sites in all breeding seasons.

The Tricolored Blackbird, a medium-sized songbird that is geographically restricted to California and small portions of adjacent states in the western United States, experienced declines in total abundance on the order of 89% from the 1930's to 1980's [6] and average colony size declines of over 60% between the 1930's and 1970's [7]. The species receives legal protection under the Migratory Bird Treaty Act and is classified as a bird species of conservation concern by the US Fish and Wildlife Service [8], and California Species of Special Concern since 1990 [9]. Additionally, it is treated as a sensitive species by the Bureau of Land Management since 1999 [10], and it has been listed on the IUCN red list of endangered species since 2006 [11]. The Tricolored Blackbird is the most colonial extant songbird in North America [12], and historically breeding colonies consisting of up to 200000 nests were recorded [13]. The species historically nested primarily in cattail (*Typha* spp.) or tule (*Schoenoplectus* spp.) marshes, but was observed to nest in a wide variety of wetland and upland habitats [13]. From the 1970's onwards the species was increasingly recorded nesting in invasive Himalayan blackberry (*Rubus armeniacus* [9], and silage crops, especially “triticale” [14], [15]. The largest recently recorded colonies have mostly occurred in triticale, a wheat [*Triticum*] x rye [*Secale*] hybrid grain grown for dairy cows, and are at risk of being destroyed when the fields are harvested before the young have fledged [5], . Recently, a federally funded program has paid farmers to delay the harvest of triticale fields occupied by breeding tricolors until after the young have fledged and left the area [16]; however, participation in this program is voluntary and not all eligible farmers participate. We previously showed that long-term (1930's to 1980's) trends in the average size of breeding colonies (numbers of birds) varied both among geographical regions and nesting substrates [7]. Cook and Toft [15] also reported that reproductive success (number of 7–9 day old chicks per nest) was greater for colonies nesting in Himalayan blackberry than for those in native cattail or tule marshes. Additionally, silage colonies had low average reproductive success because of harvest before young birds fledged [15]. Considering only non-harvested colonies, Cook and Toft [15] found that silage colonies produced more offspring per nest than cattail or tule marsh colonies. Meese [5] found no differences in reproductive success among nesting substrate types in a sample of 47 colonies. Weintraub [17] also examined whether reproductive success of colonies in silage differed from that in marsh colonies as part of a Master's thesis study, but found no differences for the 14 colonies studied. Overall, while there have been several studies of population trends (or size) and some studies of reproductive success, no study has simultaneously considered occupancy of sites and reproductive success to determine the time-averaged net value of different habitats for conservation and management.

The occupancy of breeding habitat areas, the sizes of breeding populations, and the reproductive success of breeding efforts are often readily documented, but demographic data for the rest of the life cycle are much harder to obtain. This is especially the case for species that are more widely dispersed in the non-breeding season than when breeding, such as many imperiled birds, amphibians, and aquatic insects. We often lack a good understanding of both the dispersal between populations and survival outside of the breeding season. This arises because dispersal and survival are difficult to measure (e.g., [18], [19], [20]). These data gaps are typically found in imperiled species where low abundances or restricted distribution may limit study or present ethical considerations. Consequently, conservation biologists have adopted a variety of techniques to look at habitat effects on population dynamics.

One common method is to calculate finite growth rates and apply a source-sink approach [21], [22]. However, without information about movement there is a risk of confusing habitat-specific demography with movement [23]. A source-sink approach can also be applied by using available information for reproduction in different habitats and assuming that survival has a constant value [24] and that movement does not confound measurement of finite growth rates. Such additional assumptions (about survival and dispersal) are frequently masked and increase uncertainty in the predictions made about population status. More directly, data on reproductive success is often used to identify ecological traps (e.g., [25]), although such an approach usually ignores data on the occupancy and population size in different habitats (e.g., reviewed by [26]). Of course there are studies of both source-sink dynamics and ecological traps for cases where more complete year-round data are available and movement was quantified, but this is often not the case for imperiled species. We here use a simple parsimonious method for calculating the net value for reproduction of sites in different breeding habitats by combining occupancy, abundance and reproductive data. We believe that our time-averaging approach will be useful for other species for which occupancy, abundance, and reproductive success data are available but where survival or movement data are lacking. Our approach has a more direct connection to existing data and avoids using additional assumptions to make conservation and management recommendations.

We evaluated the net value of typical sites in different breeding habitats for reproduction of Tricolored Blackbirds. Our focus was on the nesting substrate rather than the habitat surrounding nesting sites, which is used for foraging [14], and within which insect abundance at foraging locations is related to reproductive success [5]. We evaluated the net value of different nesting habitats for production of offspring by looking at the following questions: (1) Does frequency of occupancy, site extinction, or site recolonization vary by nesting substrate? (2) Does the duration of occupancy vary by nesting substrate? (3) Does reproductive success vary by nesting substrate? (4) Statewide, how frequently are breeding colonies recorded in different substrates, what are their sizes, and have their frequencies and sizes changed in recent decades? (5) Is it useful to combine the above information to obtain an overall idea of the net value of colonies in different nesting substrates in a typical year? Answering these questions allows us to provide new conservation recommendations for Tricolored Blackbirds and a methodology that is likely of broader interest to those studying the value of different breeding habitats for imperiled species.

## Methods

### Ethics

No animals were handled as a part of this study and no permits were required. The study species is not currently protected by the state or federal Endangered Species Acts which would require such permits. Some study sites are privately owned and the landowners of these sites provided access or they were viewed from nearby public rights of way without accessing the land.

### Data sources and availability

We use data from three different sources that are all publicly available:

Dataset 1. For colony occupancy and reproductive success from 2006 to 2011 we used data collected by RJM together with 2005 data collected jointly by RJM and William J. Hamilton, III. These data are already available through the public Tricolored Blackbird Portal (http://tricolor.ice.ucdavis.edu) and the explicit dataset will be made available and archived through *Dryad* (http://datadryad.org/) when this manuscript is published. This dataset includes 26 distinct sites and a total of 45 records for which reproductive success values were estimated [5].

Dataset 2. For a broader view of reproductive success we used data collected during extensive fieldwork by the late William J. Hamilton, III (WJH) between 1992 and 2005 (a few colonies were sampled jointly with RJM in 2005). These data are available in a public archive, the Knowledge Network for Biocomplexity [27]. WJH's data represent the most extensive source of information on reproductive success available for this species: it includes assessment of 128 distinct breeding sites containing colonies, and 191 records including repeated annual measurements at the same colonies, during 1992-2005. There were 2–30 colonies per year. These data up to 2000 are also discussed by Hamilton [28] but were not then formally analyzed or summarized. We have not included WJH as a coauthor since we have no way of knowing whether he would have agreed with the messages in our paper and instead directly cite the data source [27]. We did not use this dataset for occupancy analyses because it is not always clear which colonies were checked when reproductive success data were not collected.

Dataset 3. We used statewide survey data to obtain a broader view of the frequency of colonies in different breeding substrates and the size of such colonies. These data were used by Graves et al. [7] and are available in the public *Dryad* data archive ([29], file “Graves_et_al_data1.csv”).

### Empirical evaluations of reproductive success

Fieldwork generally began in late March in the southern San Joaquin Valley, where breeding commences earliest in the Central Valley, and progressed to the Sacramento Valley as the season progressed and birds move to breed again [30]. A full description of field methods are given by Meese [5], and these reflect general protocols as used by WJH. For example, the number of breeding birds in a colony was estimated either visually at the time of nesting and/or by nest sampling following the breeding season. Nest numbers were multiplied by 1.5 to estimate the number of breeding birds, which reflects that on average each male nests with two females [14]. If visual estimates of the numbers of breeding birds differed from estimates derived from direct counts of nests, the estimate derived from the direct count of nests was used because it was thought to be more accurate.

### Analyses of Occupancy, Cessation of Use, Colonization and Survival of Breeding Colonies

Breeding colonies can be treated in analogous ways to populations within a metapopulation [3] with rates of patch occupancy resulting from extinction and colonization. However, because the breeding birds using colonies do not in most cases die, we avoid referring to extinction of colonies and instead refer to “cessation of use” for breeding each year. It should however be noted that in metapopulations when a local population experiences an extinction the individuals may also have moved to another habitat patch, so the metapopulation analogy is quite strong. Analyses in this section used occupancy information from Dataset 1.

**Figure 1:**
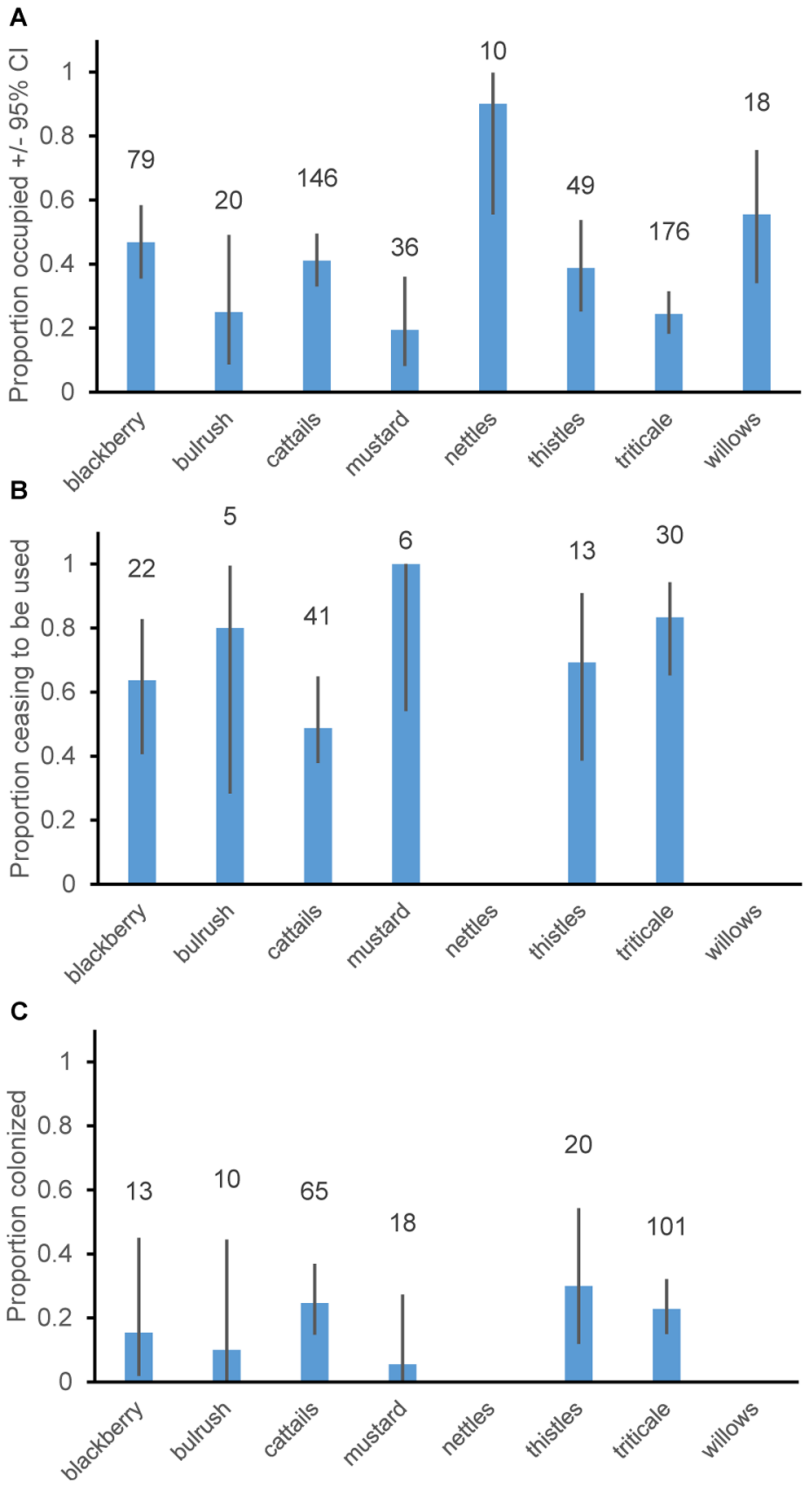
Mean proportion of breeding sites A. occupied, B. showing extinction or C. colonization per year. Numbers above bars indicate sample sizes. Error bars show 95% confidence intervals from a binomial distribution. Nettles and willows are not shown in b and c because sample sizes were less than 5.

We scored nesting sites as “occupied” when birds were present and breeding, and “unoccupied” when sites were visited but breeding birds were not found at any point during the annual monitoring period (the species' breeding season); hence sites with no information were not recorded as either unoccupied or occupied. Occupancy was analyzed using linear mixed effects models (using *lmer* in the *lme4* package in *R* [31]) with a logit link function and binomial error distribution, which are appropriate for binary data (occupied or not). In this analysis and all similar analyses, p-values (“pMCMC”) were calculated using Markov-chain Monte Carlo sampling using the function pvals.fnc from R library language [32]. Models used year as a random factor to account for repeated measures in the error structure (we also investigated using site identity as a random factor but model fit was not improved, as measured using AICc, and results were similar). We excluded substrates that had less than five total records because the sample sizes were too small to provide reliable estimates of occupancy; these included colonies situated in *Arundo donax*, buttonbush (*Cephalanthus occidentalis*), mesquite (*Prosopis* sp.), and oats (*Avena sativa*). Sample sizes for included substrates are given as the numbers above the bars in Figure 1A. We attempted to include models with the number of breeding birds as a covariate (including interactions with breeding substrate type), or the same for the area of occupied habitat prior to extinction, but neither improved model fit and we therefore do not report the results further. Because preliminary analyses indicated substantial variation in occupancy from year to year we included year as a fixed effect in the model (in addition to as a random effect to allow for repeated measures; removing the random effect of year also did not produce substantial changes in the fixed effect for year, indicating that temporal autocorrelation was weak).

A “cessation of use” event was recorded as occurring when a site was occupied by breeding birds in year *t*-1 and was not occupied in year *t*, which could have occurred either because the habitat became unsuitable (e.g., many triticale fields) or because the habitat was present and suitable, but birds no longer used it for breeding. Cessations of use were recorded as possible when a site was occupied in year *t*-1 and was monitored for nesting birds in year *t*; this procedure avoided censoring of the data. For the probability of cessation-of-use analyses we used linear mixed effects models in the same way as for occupancy listed above including covariates and year as a fixed effect. Only nesting substrate improved model fit based on delta AIC values and for brevity we do not report the factors and covariates that did not improve model fit. We included nesting substrates if there were at least 5 possible extinctions within each (sample sizes in Figure 1B), and this restriction resulted in exclusion of *Arundo*, bulrush, buttonbush, mesquite, nettles, oats, and willow substrates.

A “colonization” was recorded for sites from 2006 onwards if a site was unoccupied in year *t*-1 and became occupied in the current year *t*. Our data represent a mix of colonizations of sites that were likely unoccupied during our study and recolonizations of sites that had experienced cessations of use during our study period. Analysis was conducted in the same way as for occupancy and cessations of use, and sample sizes for included substrates are reported in Figure 1C.

We also analyzed for how many years colonies remained occupied in common breeding substrates (blackberry, cattails, thistle and triticale), and refer to this as “colony longevity.” (We use the term as a shorthand while recognizing that colonies may relocate rather than dying, hence colony longevity represents the duration of occupancy of a site.) The analysis was formerly a survival analysis using the *survreg* function from library *Survival* in *R* [33]. Preliminary analyses showed that parametric survival analyses were more informative than non-parametric (Cox's proportional hazards) analyses, and that models with a Weibull hazard function (describing instantaneous risk of death) were a significantly better fit to the data than those with an exponential hazard function. The analysis recognized that data are censored both because some colonies remained occupied by breeding birds during the breeding seasons throughout the study period and we do not know when some sites were colonized.

### Analyses of Reproductive Success

Datasets 1 and 2 were used to assess reproductive success (RS) of colonies. RS was defined as the number of chicks alive per nest at c. 7–9 days after hatching of the first egg. RS was estimated either by visual estimates or by sampling. Visual estimates of RS were derived from the estimates of the number of breeding birds obtained during monitoring and the number of fledglings observed at the end of the breeding season. Because one male breeds, on average, with two females [14], each two nests have three birds associated with them, so the product of the number of breeding birds multiplied by 2/3 (0.67) provides an estimate of the number of nests constructed. The number of young fledged divided by the estimate of the number of nests constructed yields an estimate of the number of young fledged per nest (RS).

Average reproductive success (RS) combines the numbers of offspring in successful nests with zero values that come from failed nests. Nests may fail entirely because of physical conditions (destruction during high winds, extreme temperatures, etc.) as well as predation [9]. It is therefore useful to separately consider rates of nest failure from reproduction in nests that were successful. To this end Hamilton calculated the reproductive rate for the subset of nests that were successful up to 7–9 days old, termed RSS (reproductive success of successful nests).

Because of differences in timing and observers we initially analyzed the two datasets separately. However, both visual plots and individual *lmer* models failed to find differences between the datasets, and so here we report a combined analysis. We used linear mixed effects models with colony identity as a random factor to allow for repeated measurements from individual colonies. Year, substrate and collector identity (Hamilton or RJM) were factors with fixed effects, and we also assessed year by substrate interactions but found no significant (P<0.1) effects for such interactions and do not report these results further. Collector identity (and interactions with other factors) also produced an increase in the AICc value of the model indicating that a simpler model without this variable was preferred and we therefore do not report this effect further.

### Analyses of Colonies in Different Substrates and Colony Size

We used Dataset 3 and specifically records from 1980 through 2011. We summarized the proportion of records in each breeding substrate per decade and average colony size (number of birds ln-transformed) by decade (1980–1989, 1990–1999, 2000–2009, and 2010–11). Recent colony sizes were calculated using ln(birds) per colony from 2000 to 2011 inclusive.

Recent colony sizes and reproductive success (RS) estimates from either Datasets 1 or 2 were used to estimate the total predicted production of chicks (to day 8) for average size colonies in each of the common substrates. To give an idea of variation in chick production per spring breeding per colony in each substrate we calculated a standard deviation: Standard deviations of the numbers of chicks produced were calculated as *x*.√(*s*_1_^2^+*s*_2_^2^), where *x* is the estimated number of chicks produced for a particular substrate, *s*_1_ is the proportional standard deviation for colony size (standard deviation of colony size/mean colony size), and *s*_2_ is the proportional standard deviation for reproductive success in the same substrate. Lastly, to allow for the fact that not all sites are occupied in all years we multiplied chick production by occupancy to calculate chick production across an average site of each substrate. A measure of variation could not easily be calculated for this measure but the standard deviation would likely encompass zero values (no chicks produced) for all substrates because variation in RS, colony size, and occupancy are all relatively large.

## Results

### Occupancy, Cessation of Use, Colonization and Longevity of Colonies

Average proportional occupancy of breeding sites varied widely across sites and substrates (Figure 1A). Average breeding site occupancy was significantly lower for triticale and mustard growing as a weed within grain fields, than for other breeding substrates with sufficient sample sizes (cattails, blackberry, bulrush, nettles, thistle and willow). Cattails, blackberry, bulrush, nettles, thistle and willow were similar (at P>0.1) to one-another in their levels of site occupancy (Figure 1A for differences and Tables 1, 2 for statistics). Nettle sites had higher than average occupancy, and showed significantly higher occupancy than other substrates except willows (Figure 1A and Tables 1, 2).

**Table 1:** ANOVA-style results of linear mixed effects models testing for differences in occupancy.

Fixed Effects:	SS	DF	MS	F	p	h^2^
Substrate	8.52	7	1.22	5.79	0.001	0.07
Year	4.33	6		38.46	0.003	0.04
Error	109.55	520	0.21			

The whole model adjusted R^2^-value was 11%. Random effects were: Year (Intercept) variance  = 0.11423, standard deviation  = 0.33798, from 534 observations in 7 groups (years). Effect size is given as the proportion of variance explained by explanatory variables, partial eta-squared (h^2^) = (SS_effect_)/(SS_effect_+SS_error_).

**Table 2:** Parameter values from linear mixed effects models testing for differences in occupancy.

Parameter type	Group	Parameter	SE	z	p
Mean	cattails, 2005	0.058	0.30	0.19	0.85
difference in mean	mustard	-1.11	0.46	−2.40	0.02
difference in mean	blackberry	0.34	0.29	1.16	0.25
difference in mean	bulrush	−0.78	0.55	−1.42	0.16
difference in mean	nettles	2.98	1.08	2.74	0.006
difference in mean	thistle	−0.02	0.35	−0.06	0.95
difference in mean	triticale	−0.82	0.25	−3.32	0.001
difference in mean	willow	0.69	0.53	1.31	0.19
difference in mean	2006	−0.44	0.38	−1.17	0.24
difference in mean	2007	−0.37	0.39	−0.96	0.34
difference in mean	2008	0.12	0.35	0.34	0.73
difference in mean	2009	−0.34	0.36	−0.96	0.34
difference in mean	2010	−0.48	0.38	−1.27	0.20
difference in mean	2011	−1.23	0.36	−3.46	0.001

The mean value of logit-transformed occupancy is given for cattails in 2005, and then other rows of the table give the difference (in logit-transformed mean occupancy) from this value for the groups indicated.

The rate of cessation of breeding at sites that were used for breeding in previous years was generally frequent, with an average of 66% of sites per year ceasing to be occupied by breeding birds. This rate was significantly higher for triticale fields (83% of sites per year) than for cattail sites (49%; Figure 1B; Tables 3, 4). Data on cessation of use of breeding sites were sparse for blackberry, bulrush, mustard, nettle and willow sites (Figure 1B), which might account for a lack of any statistical differences (at P<0.1) in the frequency of cessation of use of sites in these substrates compared to other substrates. Although with a small sample size it is noteworthy that like triticale sites, mustard sites showed a high average rate of extinction (100%). This likely reflects either that annual crops were not planted in the same place each year or that weeds in such fields were removed by herbicide application, forcing extinction through a lack of habitat in the form of both the crop itself and mustard as a weed within such crops.

**Table 3:** ANOVA-style results of linear mixed effects models testing for differences in the proportion of colonized sites where occupancy for breeding ceased per year.

Fixed Effects:	SS	DF	MS	F	P	h^2^
Substrate	2.93	5	0.59	2.82	0.019	0.11
Error	23.07	111	0.21			

Random effects were: Year (Intercept) variance = 3.8×10^−13^, standard deviation = 6.2×10^−7^, from 117 observations in 6 groups (years). Effect size is given as the proportion of variance explained by explanatory variables, partial eta-squared (h^2^) = (SS_effect_)/(SS_effect_+SS_error_).

**Table 4:** Parameter values from linear mixed effects models testing for differences in the proportion of colonized sites where occupancy for breeding ceased per year.

Parameter type	Group	Parameter	SE	z	p
Mean	cattails	−0.049	0.31	−0.16	0.88
difference in mean	mustard	16.6	1615	0.01	0.99
difference in mean	blackberry	0.61	0.54	1.12	0.26
difference in mean	bulrush	1.44	1.16	1.24	0.22
difference in mean	thistle	0.86	0.68	1.27	0.20
difference in mean	triticale	1.66	0.58	2.85	0.004

The mean value of logit-transformed proportion of sites with cessation of breeding is given for cattails, and then other rows of the table give the difference (in logit-transformed mean proportion) from this value for the groups indicated.

For the six substrates with calculable rates at which they ceased to be used for breeding, these rates were strongly negatively correlated with occupancy (Pearson's r = −0.87, P<0.025 in a 1-tailed test). The overall pattern is that the two temporary habitats, triticale and mustard, showed lower occupancy (Figure 1A) and higher observed rates of cessation of use (Figure 1B) than other types of breeding site. This likely reflects habitat loss either through herbicide use on weeds that Tricolored Blackbirds frequently nest in (e.g., mustard) or because of crop rotations. The two substrates for which rates of cessation of use could not be calculated (because n<5) were nettles and willows, both of which showed very high occupancy (Figure 1A) and thus experienced very few cessations of use.

Colonization rates were generally low, with only 21.1% of sites per year being colonized each year. *LMER* models showed no significant difference (at P<0.1) for any substrate or overall (Tables 5, 6). Across the full suite of sites for which we had occupancy data the low colonization rates (21%/year) relative to cessation rates (66% sites/year) could either reflect a declining (nonequilibrium) metapopulation or that colonizations are under-recorded.

**Table 5:** ANOVA-style results of linear mixed effects models testing for differences in the proportion of vacant sites with colonizations per year.

Fixed Effects:	SS	DF	MS	F	P	h^2^
Substrate	0.86	5	0.17	1.01	0.41	0.02
Error	37.6	221	0.17			

Random effects were: Year (Intercept) variance = 0.004, standard deviation = 0.066, from 227 observations in 6 groups (years). Effect size is given as the proportion of variance explained by explanatory variables, partial eta-squared (h^2^) = (SS_effect_)/(SS_effect_+SS_error_).

**Table 6:** Parameter values from linear mixed effects models testing for differences in the proportion of vacant sites colonized per year.

Parameter type	Group	Parameter	SE	z	P
Mean	cattails	−1.12	0.29	−3.87	0.001
difference in mean	mustard	−1.72	1.07	−1.61	0.11
difference in mean	blackberry	−0.59	0.82	−0.71	0.48
difference in mean	Bulrush	−1.08	1.09	−0.99	0.32
difference in mean	Thistle	0.27	0.57	0.48	0.63
difference in mean	triticale	−0.10	0.37	−0.27	0.79

The mean value of logit-transformed proportion colonized is given for cattails, and then other rows of the table give the difference (in logit-transformed mean proportion colonized) from this value for the groups indicated.

**Figure 2:**
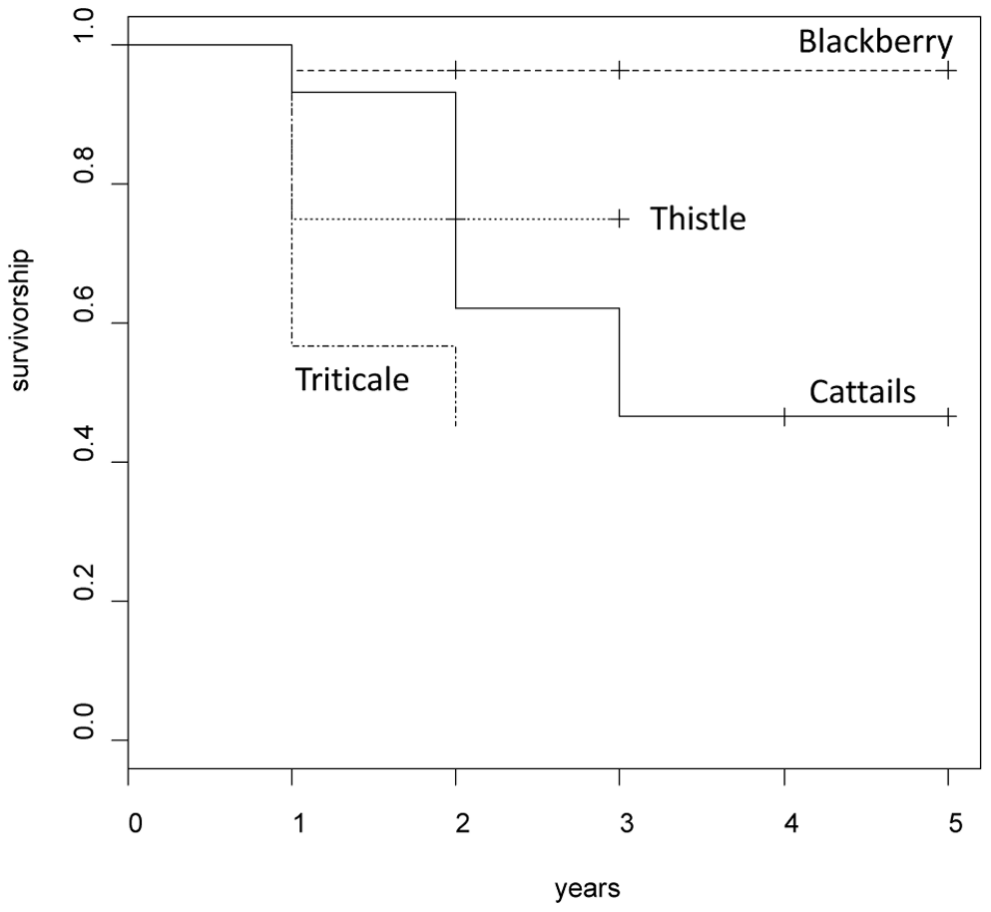
Survivorship for breeding colonies in different substrates. The vertical crosses (plus symbols) indicate that datapoints were constrained by censoring of the data. Note that for Blackberry there was only one non-censored event and so the survivorship values are limited by sample size and are likely not reliable.

Analysis of the numbers of years for which sites remained in use by breeding colonies using survival analysis revealed that the slope of survivorship versus age of colonies declined with colony age (scale parameter = 0.436, Table 7). Hence colonies that were occupied for more than 1 year were less likely to cease being occupied during their second year than their first year (Figure 2). Continued use of sites in cattail marshes was more likely than for triticale sites (Figure 2, Table 7). This accords with the high per year cessation-of-use rates of triticale colonies compared to cattail marsh colonies (Figure 1B, Tables 3, 4). Survivorship slope declining less sharply in older colonies can most clearly be seen in cattail colonies (Figure 2), whereas triticale colonies frequently ceased to be used after one year, and sample sizes were small because there were few uncensored records for blackberry and thistle colonies.

**Table 7:** Results of parametric survival analysis for breeding colonies using a Weibull hazards function.

Parameter type	Group	Parameter	SE	z	p
Mean	Cattails	1.355	0.169	8.03	0.001
difference in mean	blackberry	0.582	0.476	1.22	0.21
difference in mean	Thistles	−0.334	0.301	−1.11	0.27
difference in mean	Triticale	−0.805	0.202	−3.99	0.001

The model was significantly preferred over an intercept-only model (Chi-squared = 22.44 with 3 degrees of freedom, p<0.001). Weibull scale parameter = 0.436. The mean value of survival is given for cattails, and then other rows of the table give the difference from this value for the groups indicated.

### Reproductive Success

**Figure 3:**
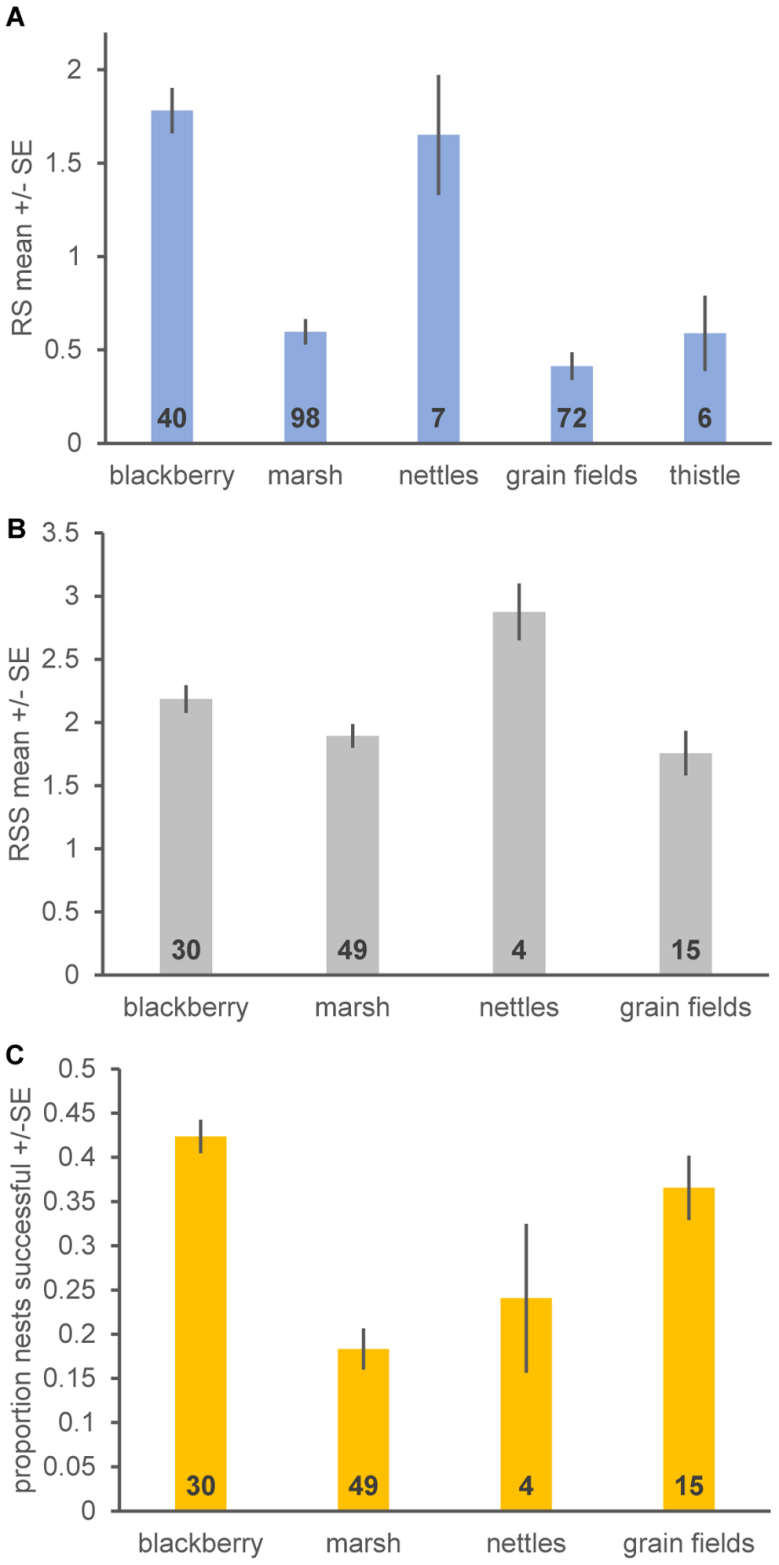
Reproductive success estimates for different breeding substrates. Estimates of **A**, reproductive success (RS), defined as the average number of chicks per nest at c. 8 days after the first egg hatched, **B** reproductive success of nests that were successful in rearing some young to day 8 (RSS), and **C** the proportion of nests that were successful in rearing some young to 7–9 days-old. Data in **A** come from Hamilton and RJM, and those in **B** and **C** come from Hamilton. Bars indicate standard errors. Numbers inside the base of bars indicate sample sizes (colonies x years, reflecting that these data include some repeated measurements).

Reproductive success (RS) varied substantially among nesting substrates, and for habitats with at least 5 RS values substrate accounted for 59% of the variation in RS values (Tables 8, 9). Himalayan blackberry colonies had a greater average reproductive success than marshes, grain fields, and thistle habitats (Tables 8, 9; Figure 3A). The sample size for RS estimates from nettles was low (Figure 3A) and statistically there was no difference from other substrates (Tables 8, 9), but RS values were high and grouped together with blackberry. There were only 4 RS estimates from colonies in willows and the RS values were low and seemed similar to thistle, marsh and grain field colonies. The analysis reported in Tables 8, 9 did not find any significant (P<0.1) effects of observer (Hamilton or Meese) or year on RS values and so the above results represent a compilation of the datasets. Colony size (estimated number of birds) did not have any statistical effects on RS in the linear mixed effects models, nor did colony area (square meters) in the Meese data (and was not collected for the Hamilton data).

**Table 8:** ANOVA-style results for linear mixed effects model analyses of reproductive success (RS) for both the Hamilton and Meese datasets.

Fixed Effects:	SS	DF	MS	F	p	h^2^
Substrate	58.3	4	14.6	31.6	<0.001	0.59
Error	98.8	214	0.46			

The analysis was limited to breeding substrates with at least 5 measurements. Collector identity and year of collection were removed in model simplification and are not reported further. Effect size is given as the proportion of variance explained by explanatory variables, partial eta-squared (h^2^) = (SS_effect_)/(SS_effect_+SS_error_). Random effects were: Colony identity (intercept) variance = 0.136, standard deviation = 0.368, from 219 observations in 138 groups (colony identities).

**Table 9:** Parameter values from linear mixed effects model analyses of reproductive success (RS) for both the Hamilton and Meese datasets.

Mean	Blackberry	1.78	0.12		15.2	0.0001
difference in mean	Marsh	−1.16	0.14		−8.25	0.0001
difference in mean	Nettles	−0.10	0.29		−0.34	0.66
difference in mean	Grain fields	−1.32	0.15		−8.46	0.0001
difference in mean	Thistle	−1.19	0.30		−3.93	0.0001

The analysis was limited to breeding substrates with at least 5 measurements. P-values (“pMCMC”) were obtained using Markov-chain Monte Carlo sampling using the function pvals.fnc from R library language [32]. Collector identity and year of collection were removed in model simplification and are not reported further. The mean value of reproductive success is given for marsh habitat, and then other rows of the table give the difference from this value for the groups indicated.

Reproductive success results in part from complete failure of nests, from sampled nests in which eggs were never laid, and in part from reduced numbers of chicks in nests that survive to the time of recording (day 7–9). Figure 3C shows that a low proportion of nests was successful at rearing young in marsh habitats compared to those in Himalayan blackberry and grain field sites. Stinging nettle sites appeared intermediate and variable (likely because of small sample sizes; Figure 3C). Interestingly nesting substrate accounted for only 15% of variance in RSS compared to the 54% in RS, indicating that nesting substrate had a more predictable effect on whether nests failed or succeeded in raising some chicks rather than on the numbers of chicks produced. As with RS, RSS was relatively high for Himalayan blackberry colonies (Tables 10, 11, Figure 3B). Grain fields had lower RSS than Himalayan blackberry colonies, and nettle colonies had higher RSS than Himalayan blackberry colonies (and grain fields; Tables 10, 11, Figure 3B). Marsh colonies had lower reproductive success than Himalayan blackberry colonies but significance was marginal (pMCMC = 0.056; Tables 10, 11), reflecting small sample size for RSS from marshes. RSS for marsh colonies was similar to that from grain field colonies (Figure 3B).

**Table 10:** ANOVA-style results for linear mixed effects model analyses of reproductive success of nests that were successful in rearing at least one chick to day 8 after first egg hatch (RSS) for the Hamilton dataset.

Fixed Effects:	SS	DF	MS	F	p	h^2^
Substrate	5.53	3	1.84	4.56	0.005	0.15
Error	37.6	93	0.40			

The analysis was limited to breeding substrates with at least 5 measurements. Effect size is given as the proportion of variance explained by explanatory variables, partial eta-squared (h^2^) = (SS_effect_)/(SS_effect_+SS_error_). Random effects were: Colony identity (intercept) variance = 0.006, standard deviation = 0.08, from 97 observations in 74 groups (colony identities).

**Table 11:** Parameter values from linear mixed effects model analyses of reproductive success of nests that were successful in rearing at least one chick to day 8 after first egg hatch (RSS) for the Hamilton dataset.

Parameter type	Group	Parameter	SE	t	pMCMC
Mean	Blackberry	2.19	0.12	18.681	0.0001
difference in mean	Marsh	−0.29	0.15	−1.958	0.056
difference in mean	Nettles	0.69	0.34	2.035	0.046
difference in mean	Grain fields	−0.43	0.20	−2.124	0.038

The analysis was limited to breeding substrates with at least 5 measurements. The mean value of RSS is given for marsh habitat, and then other rows of the table give the difference from this value for the groups indicated. P-values (“pMCMC”) were obtained using Markov-chain Monte Carlo sampling using the function pvals.fnc from R library language [32].

### Frequencies of Colonies in Different Substrates and Colony Size

**Figure 4:**
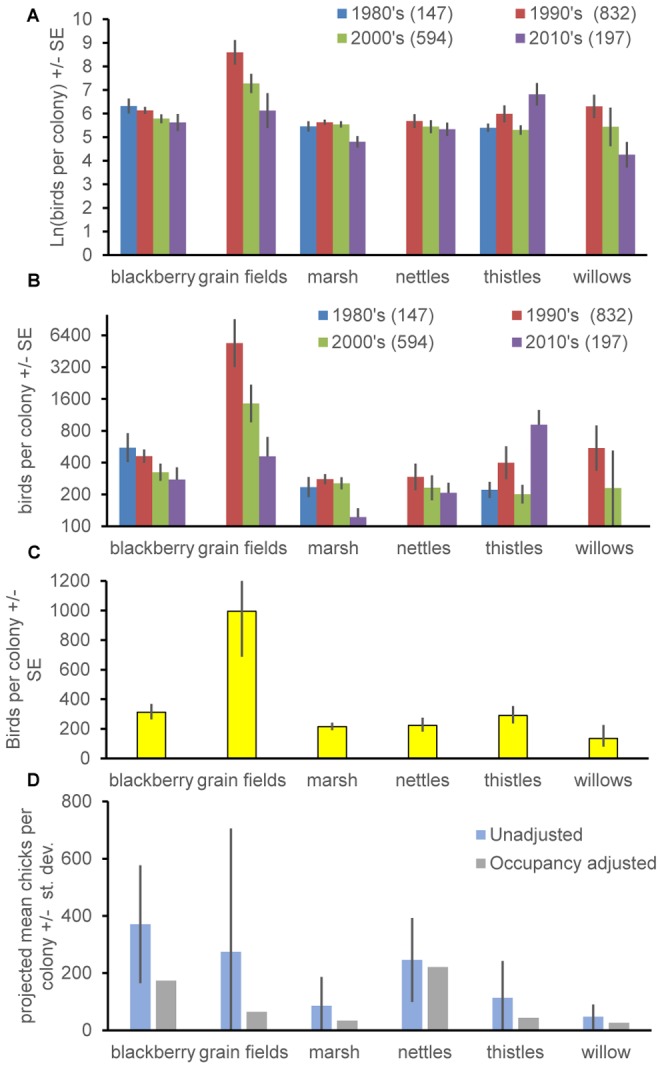
Frequency of colonies, colony size and projected net chick production per colony. **A**. Proportion of colonies in different substrate types by decade, with total sample sizes in parentheses. **B**. Size of colonies in different substrates by decade (color key same as in a). **C**. Size of recent (2000–2011) colonies. **D** Projected number of chicks produced per colony of average size using reproductive success estimates from Figure 3A and also the same estimates adjusted for the fact that an average site is not occupied in every year (using analyses in Figure 1A). In B and C error bars show +/− 1 SE to facilitate comparison, whereas in D error bars are +/− 1 standard deviation to give an idea of variation. Error bars (standard deviations) are not readily calculable for the occupancy-adjusted projected chicks per colony but likely overlap zero because they represent the summation of at least 3 sources of error (compared to 2 for the other two estimates in D).

Figure 4A shows that colonies were most frequent in marsh habitats (cattails and bulrush) followed by blackberries and thistles. Records in grain fields (primarily triticale but also mustard within triticale) have grown steadily to represent 8.6% of colonies in 2010–2011. The proportion of records grew through time for both nettles (reaching 10.2% of records in 2010–11) and thistle (12.7% of records in 2010–11). Conversely the proportion of records in marsh habitats declined steadily through time (Figure 4A), from 51.7% in the 1980's to 33% in 2010–11. With the exception of thistle colonies, the average size (number of birds) of colonies in common substrates was smaller in 2010–11 than in previous decades (Figure 4B). The decline was most dramatic for grain crops (Figure 4B). For the period 2000 to 2011 inclusive, representing recent records (without putting too much emphasis on 2010–11) Figure 4C shows average colony sizes. Grain field colonies were by far the largest on average size, with a mean of 995 birds. Other colonies on average had 312 birds in blackberry, 290 for thistle (and milk thistle, *Silybum marianum*), 224 birds for nettle, 215 birds in marsh substrates and the few willow sites were smallest of all (135 birds).

Predictions of the numbers of chicks that would have been produced by average size colonies were in general highly variable, reflecting that both the RS estimates and colony size estimates were also variable. Putting together RS estimates and average (2000–2011) colony sizes leads to the prediction that blackberry and grain field colonies produced the most chicks on average (Figure 4D). This was followed by stinging nettle colonies and then thistle colonies (Figure 4D). Marsh sites produced smaller numbers of chicks on average but they were still about twice as productive as willow sites (Figure 4D). Incorporating occupancy into our analysis across the years shows that nettle sites were the most productive (with a mean of 221 chicks per site per year; Figure 4D) because they have high occupancy, followed by blackberry sites (174 chicks/site/year). (An average grain field in an average year produced 65 chicks, but this figure is not very relevant because grain fields are generally not conserved from year to year). Thistle sites produced an average of 44 birds/site/year, and surprisingly marsh sites produced an average of only 34 birds/sites/year reflecting that their occupancy was low. The few willow sites produced an average of 26 birds per year. Clearly conserving triticale (grain) fields when they are occupied is especially valuable and this is possible because the habitat is not permanent. Apart from this, considering occupancy leads to the prediction that average nettle sites are disproportionately important in chick production, as are blackberry sites, whereas thistle sites are less important and marsh sites are close to least important of the nesting substrates commonly used by Tricolored Blackbirds.

## Discussion

Our analyses demonstrate a simple direct method for combining data on breeding site occupancy, breeding population sizes and reproductive success to calculate the net metric for the value of different habitats for reproduction. In our case because we had time series of occupancy values for each site, we calculated time-averaged values for reproductive success, but such calculations could also be made using one-time (snapshot) estimates of occupancy, abundance and reproduction. Such direct calculations avoid making additional assumptions about survival (outside of the breeding season) and dispersal that would be required to apply a source-sink model (e.g., [21]) to species where we have data only on breeding populations. We believe that such calculations would also benefit studies of other imperiled bird species, as well as other taxa where we can readily obtain data only on breeding success and breeding populations because individuals are more widely dispersed when not breeding. It is surprising that previously (as far as we can determine) such an index has not been described. Our calculations assume that there is turnover of occupancy in sites, as is usually the case in fragmented populations and metapopulations [20].

Calculation of the average number of offspring produced per site in an average year provides a method of assessing the conservation value of different breeding substrates (Figure 4D). An assessment of the components making up this number, like that in Table 12, helps us understand multiple components of the value of colonies, in particular breeding substrates, average breeding colony size, occupancy, nest failure rates, and numbers of young surviving to a given point in time. It is useful to consider each substrate in turn, which we do below from highest to lowest time-averaged total estimated number of chicks produced for an average colony.

**Table 12:** Summary of the differences between colonies in different substrates.

Substrate	Occupancy	Colony longevity	RS	RSS	Frequency of colony failure	Frequency of substrate type	Colony size 2000–2011	Predicted long-term average site productivity
Himalayan blackberry	0	0	+	0	-	0	0	+
Marsh	0	0	0	0	0	+	0	-
Nettles	+	+	+	+	-	-	0	+
Grain fields	-	-	-	0	+	-	+	0
Thistle	0	0	0	0?	0?	0/-	0	0
Willows	0/+?	+?	0?	0?	0?	-	-	-

Colony longevity was inferred from a mixture of survival analyses and extinction analyses. + indicates above average, 0 indicates average, and - indicates below average. A question mark indicates that sample sizes were especially small.

We showed the following for Tricolored Blackbirds: (1) The frequency of occupancy and site extinction (cessation of use) varied substantially among different nesting substrates, but we found no differences in rates of site recolonization by nesting substrate. (2) As predicted by different frequencies of extinction (cessation of use), the duration of occupancy varied among nesting substrates. (3) Reproductive success showed substantial differences among nesting substrates. (4) Statewide average sizes of breeding colonies in different substrates and frequency of occurrence in different substrates (number of sites) changed through time. The pattern was generally with traditional marsh sites being used less frequently and supporting smaller colonies relative to colonies in native nettles and invasive thistles. Himalayan blackberry colonies are fairly typical in size, occupancy and longevity, and occur with a typical frequency. However Hamilton's data indicate that these colonies have a low failure rate and a higher reproductive success and lower rates of nest failure than other breeding substrates (Figure 3). Consequently long-term breeding productivity of an average blackberry site is expected to be high (Figure 4D). This accords with the findings of Cook and Toft [15], who recorded higher reproductive success for nests in Himalayan blackberry than in other substrates. Unfortunately, Himalayan blackberry is a high risk nonnative invasive species [34] and so it cannot be planted as a component of many federally-funded conservation programs and is frequently removed or attempted to be removed [35]. Himalayan blackberry is problematic because of competition with native plant species, reducing soil moisture and as a potential fire hazard [34]. As Cook and Toft [15] point out there is a conflict between this invasive weed and habitat for Tricolored Blackbirds.

Stinging nettle sites had high occupancy, longevity and reproductive success, and low rates of failure. Consequently nettle sites on average have high long-term breeding productivity (Figure 4D). Stinging nettle sites are however infrequent in occurrence (Figure 4A). Previous studies of reproductive success have lacked sufficient data to evaluate nettle sites. Stinging nettles are native and could be planted to provide breeding substrate for Tricolored Blackbirds but require a reliable supply of fresh water before and during the tricolor's breeding season so may be limited as a conservation tool due to water scarcity.

Marsh colonies (cattails and bulrushes) are the most frequent colony type yet are average compared to other colony types in all aspects measured, including occupancy, longevity, size, reproductive success, and rate of nest failure. The lack of any more positive aspects to marsh sites relative to other colony types makes the net breeding productivity of an average site relatively low (Figure 4D), and consequently their conservation value for Tricolored Blackbirds is more limited than blackberry and nettle sites. Cook and Toft [15] found similar results. Tricolored blackbirds prefer marshes containing vegetation that is young, lush, and rapidly growing, and will avoid older cattail and bulrush marshes containing much thatch and many lodged, dead stems. Hence, marsh management consisting of actions designed to remove old, dead stems and encourage regrowth of new vegetation is needed to promote the use of marsh habitats. In most cases, annual burning is required to rejuvenate marshes and to provide the conditions preferred by breeding tricolors. Water levels are also critical to reducing predator access, as raccoons (*Procyon lotor*), the tricolor's most serious predator in freshwater marshes, prefer to wade than to swim, and typically will not cross deep channels around the perimeter of cattail stands. To this end, the management of marshes for Tricolored Blackbirds by private duck clubs is a potentially important component of a comprehensive conservation strategy since Tricolored Blackbirds and a host of wetland-dependent species may benefit from the springtime availability of water.

Cereal grain fields, including triticale, wheat, and mustard (*Brassica* spp.) growing as a weed within such fields, have since the 1980's held by far the largest colonies (Figure 4C) but have relatively low net reproductive success because of a high rate of colony destruction through harvest (Table 12; Figures 3, 4D). Triticale colonies are frequently destroyed through harvest because the crop ripens before the young fledge and farmers harvest their fields when the seed heads reach maturity [14]. The fact that grain field occupancy is low (even replanted sites are frequently not reused; Figure 1A) and reproductive success is moderate means that a more dynamic conservation strategy is needed (and used) for cereal grain crops; temporary large breeding colonies in grain crops are best targeted when they are present. Cook and Toft [15] also found that colonies in triticale crops that were not harvested had relatively high reproductive success (mean RSS = 1.0), but not as high as the larger dataset used here (mean RSS = 1.76; Figure 3B). Overall the findings for triticale crops accord with both the recommendations of the Tricolored Blackbird Working Group [16] and the use of federal funds to encourage farmers to volunteer to delay harvest of triticale crops containing Tricolored Blackbird breeding colonies. It is not clear that a more permanent preservation of repeatedly planted sites are especially valuable for Tricolored Blackbird conservation because they have a low occupancy by breeding colonies through time. While we recognize that birds breeding in farmers' fields contains great inherent risks, given the relatively large number of birds that breed in grain fields adjacent to dairies and the absence of nearby alternative nesting substrates, it is essential as a core component of a comprehensive conservation strategy that all of these colonies be protected until the young have fledged. In the longer term, additional protected breeding substrates must be provided to give birds secure nesting habitats while ensuring the farmer's right to harvest his crop.

Colonies in thistle (e.g., bull thistle, *Cirsium vulgare* and milk thistle, *Silybum marianum*) substrates are relatively infrequent but are typical in occupancy, longevity, reproductive success (but data on failure rates are lacking), and size; consequently they have a typical net long-term productivity per site that is similar to that for grain fields despite the much smaller colony size in thistle sites. In one year (2010) the largest known colony was in milk thistle and had an estimated 83000 birds, which also illustrates that year-to-year variation is high. Again there is the problem that both of these plant species are invasive, although the impacts of milk thistle are limited [34]. Hence a conservation strategy preserving sites and maintaining vegetation type would likely be effective for thistle and milk thistle sites, but nettle substrate is both native and more valuable. Lastly, although data were sparse for willow sites, colonies were small and infrequent, making their net breeding productivity relatively low and consequently their conservation value also low.

A question that arises from our analyses is what is the mechanism (or mechanisms) by which nesting substrate influences reproductive success. Meese [5] showed a clear correlation between insect abundance (food) in habitats around nesting colonies and RS of those colonies in the same year, and only colonies with abundant insects were successful at rearing some young. Meese's analysis produced a correlation between ranked values of 0.74, and hence accounted for 54% of the variation in ranked RS values. It is possible that nesting substrates reflect neighborhood insect abundances, although other effects are also possible. In our analyses breeding substrate accounted for 54% of variation in RS (the same as insects in Meese's study [5]). More importantly, breeding substrate accounted for only 15% of variation in RSS (reproductive success of successful nests), which is consistent either with nesting substrate having greater predictive ability for whether nests succeed or fail, rather than in the number of chicks that produced, or with there being a threshold effect such that RS is more likely to become zero in certain breeding substrates. Beedy, and Beedy and Hamilton [9], [14] report that the basic requirements for successful breeding are nesting substrates that are protected by virtue of being flooded, or possess thorny or spiny leaves or stems, and that occur in proximity to foraging habitats. Other studies have reported colony failures because of both predation (e.g., [5], [9], [17], [36], [37]), loss of standing water in marsh sites (which also may increase predation, (e.g., [38])) harvest of grain crops (above), and habitat destruction (e.g., [39])). Hence we expect that breeding substrate could have a direct role on colonies by reducing rates of predation. Large losses from colonies have been reported due to predation by Black-crowned Night-herons (*Nycticorax nycticorax*), Cattle Egrets (*Bubulcus ibis*), White-faced Ibis (*Plegadis chihi*), Common Ravens (*Corvus corax*), Coyotes (*Canis latrans*) [5],[9],[17],[36],[27]. Avian predators can access nests even in flooded habitats, whereas terrestrial predators can more easily access dried out marshes or terrestrial habitats. Thorny and spiny terrestrial habitats and nests sufficiently far above the ground (e.g., 3-m above the ground in willows [9]) may offer some protection from most predators. The degree to which different habitats differ in predation rates needs more systematic study (as also suggested by [9]). In the central coast of California numbers of some predatory herons and egrets have increased since 1991 [40], and although data are sparse for the Central Valley of California (the area containing most Tricolored Blackbirds), some species have increased nationally (see references in [40]). Beyond the obvious effect of harvesting of colonies in grain fields, the relative extent of disturbance in different habitats requires further evaluation. The kinds of effects are exemplified by Meese [39] who reported a Himalayan blackberry colony that was defoliated causing the birds to abandon the site, and two milk thistle colonies that were destroyed by cutting. Weintraub [17] also reported that some more terrestrial sites (Tamarisk and mesquite) were only used when they were flooded, and hence flooding of sites and conditions more generally might affect site at the time of habitat selection, prior to nesting.

Our results in conjunction with Meese's [5] study of food availability in areas surrounding breeding sites indicate that we need to disentangle the effects of nesting substrate, habitats available within the foraging area of breeding Tricolored Blackbirds, and food availability. All three of these things may be correlated or they may be independent. They may also not be mutually exclusive. The problem of analyzing the foraging habitats is made difficult by birds traveling up to 5 to 9km from their nesting sites [5],[14],[41],[42], but as Hamilton and Meese [43] point out, only a small fraction of the total possible area may be suitable foraging habitat. Beedy [9] also suggested investigation of foraging habitat availability near colonies, and habitat selection. Investigating habitat selection mechanisms and relative use of different substrates is particularly difficult but it may be that year-to-year variation in the availability of different habitats would provide the best evidence of (correlative) shifts in habitat use, perhaps in conjunction with potential driving variables like rainfall (e.g., [17]).

The suggested conservation strategies for Tricolored Blackbirds of providing alternative habitats and luring birds from grain fields [9] are consistent with our findings of the use and reproduction of different habitats. However, stinging nettle sites seem like the most widely used native habitat type that is productive and may represent the best opportunity for native habitat creation, conservation and restoration. The management of cattail marshes, as the most frequently used marsh type, needs more research linking marsh state to nest success and predation, and may represent a realizable habitat management strategy because protected lands often contain wetland areas. In the short term the voluntary payment of farmers to encourage them to delay harvest of grain crops (triticale) for silage needs to be continued and other strategies of alleviating pressures such as water restrictions on dairy farms that regularly support Tricolored Blackbird merit investigation by management agencies.

The lack of balance between cessation of use (“extinction”) and colonization of breeding sites 66% sites/year vs. 21% sites/year reflects that Meese's fieldwork took place during 2005–2011 and that 2007 onwards was a period when reproductive success was chronically low [5]. Population sampling has been more thorough than ever and so these data are unlikely to represent changes in sampling effort. Statewide surveys suggested populations declined by 35% between 2008 and 2011 [44],[45], and declines in average colony size are apparent over a longer period in Figure 4B. Both colony sizes and declines in occupancy during 2005–2011 are consistent with a metapopulation that is in steep decline. However, the timespan is short and it remains to be determined whether the 2014 survey (and beyond) will show sustained declines. Neither total abundances nor colony sizes were correlated with rate of (re)colonization of sites or probability of cessation of use of sites for breeding (or reproductive success, RS). In this way the system does have the feedbacks expected of a typical metapopulation [4], which might reflect the species being in decline during 2005–2011: our analyses looked at these factors in conjunction with nesting substrate types so heterogeneity in substrates is unlikely to mask such a pattern.

Future studies should attempt to (1) estimate rates of predation from site to site and between substrate types, which is made complicated by the large number of sites needed; (2) understand whether nesting substrate type is linked to landscape composition and food availability, or whether these are independent drivers of reproductive success; (3) evaluate whether marsh management for Tricolored Blackbirds results in predictable increases in RS, abundance and occupancy; and (4) investigate the potential for habitat creation and restoration involving stinging nettles. There is an urgent need to also ascertain whether the species is continuing in sharp decline across all habitat types and to discover the causes of this decline beyond those identified here. Climate, agricultural changes, and land-use changes all merit investigation as potential causes.
